# Prospective study of predictors of continuous smoking abstinence after hospital discharge

**DOI:** 10.18332/tpc/217012

**Published:** 2026-04-20

**Authors:** Avery Roberson, Kinsey Pebley, K. Michael Cummings, Vincent Talbot, Asia A. Bliss, Stephanie Stansell, Benjamin A. Toll

**Affiliations:** 1 Department of Psychiatry and Behavioral Sciences, Medical University of South Carolina, Charleston, United States; 2 Department of Public Health Sciences, Medical University of South Carolina, Charleston, United States; 3 TelASK Inc, Mount Pleasant, United States; 4 Department of Population Health, Medical University of South Carolina, Charleston, United States

**Keywords:** smoking cessation, cigarette smoking, tobacco control, hospital discharge

## Abstract

**INTRODUCTION:**

Smoking after discharge from the hospital increases the risk of adverse health outcomes such as unplanned hospital readmissions. This prospective study explores predictors of self-reported continuous smoking abstinence in adult patients 6 weeks after hospital discharge.

**METHODS:**

The study population includes 985 adult currently smoking patients, admitted to the hospital between March and December 2021, who were discharged home following their hospital stay and followed up 6 weeks later to reassess smoking status. Electronic health records (EHRs) were used to measure potential predictors of smoking abstinence and included patient demographic factors (age, gender, race, insurance status), clinical characteristics (i.e. length of hospital stay, Charlson Comorbidity score, and other comorbidities such as alcohol or other substance use disorders), and exposure to the hospital’s tobacco treatment program (TTP).

**RESULTS:**

Among the 985 patients reached for follow-up, 21.1% reported continuous abstinence from cigarette use 6 weeks after hospital discharge. In adjusted analyses, significant predictors of smoking abstinence included being male (adjusted odds ratio, AOR=1.5; 95% CI: 1.0–2.1), older age (AOR=1.02; 95% CI: 1.0–1.04), fewer years smoking (AOR=0.97; 95% CI: 0.95–0.99), and longer hospital stay length (AOR=1.42; 95% CI: 1.1–1.8). A diagnosis of substance use disorder was also associated with lower odds of abstinence (AOR=0.37; 95% CI: 0.2–0.7) while exposure to the TTP was not associated with smoking abstinence at follow-up.

**CONCLUSIONS:**

Patients requiring longer hospitalizations were more likely to report smoking abstinence after discharge.

## INTRODUCTION

Continued smoking after discharge from the hospital increases the risk of unplanned hospital readmissions^1^. Hospitalization provides an opportunity for patients who currently smoke to stop smoking and remain smoke-free after discharge^2,3^. Patients are not allowed to smoke during hospitalization, and today most inpatient facilities have the potential to provide patients with access to therapies to address temporary nicotine withdrawal symptoms (i.e. the ability to prescribe and provide nicotine replacement therapy [NRT] while admitted and upon discharge), which could translate to extending cessation after hospital discharge^3^. Previous studies have described the positive impact of tobacco cessation programs delivered during hospitalization, at the time of discharge, and immediately after discharge from the hospital^2-14^.

In 2012, the Joint Commission recommended that hospitals screen all patients for current tobacco use, provide smoking cessation treatments to patients who smoke during hospitalization and at the time of discharge, and follow up with patients within a month after hospital discharge^15^. Several years ago, the Medical University of South Carolina (MUSC) healthcare system implemented a tobacco treatment program (TTP) for multiple inpatient facilities modeled after the Joint Commission guidelines^16-18^. Briefly, patients received bedside consultation and an automated phone call seven days after discharge, allowing patients the opportunity to connect with the South Carolina Tobacco Quitline (SCTQ) where they can receive additional counseling and access to free smoking cessation medications. Despite the intervention, a follow-up survey of patients referred to the TTP conducted six weeks after patients were discharged home found that 79% were smoking again^18^. Only a few studies have evaluated predictors of smoking abstinence after hospital discharge^19,20^.

The prospective study was designed to explore a wide range of patient characteristics and clinical predictors of self-reported continuous smoking abstinence 6 weeks after hospital discharge among patients referred to the MUSC TTP. In addition, this study evaluates how exposure to different elements of the MUSC TTP and reported use of smoking cessation medications after hospitalization affected self-reported smoking abstinence.

## METHODS

### Study population and design

This prospective study explores predictors of self-reported continuous smoking abstinence in adult patients 6 weeks after hospital discharge. Electronic health records (EHRs) were used to measure potential predictors of smoking abstinence and included patient demographic factors (age, gender, race, insurance status), clinical characteristics such as length of hospital stay, Charlson Comorbidity score, other comorbidities such as alcohol or other substance use disorders, and exposure to the hospital’s TTP.

The study population includes 985 adult current smoking patients admitted to the hospital between March and December 2021 who were discharged home following their hospital stay and followed up 6 weeks later to reassess smoking status. To be included in our sample, patients had to have been hospitalized for at least 48 hours, be discharged to their home, have a working phone number, speak English, complete our Research Electronic Data Capture (REDCap) follow-up telephone survey, and acknowledge in the follow-up survey that they had been a current smoker at the time of admission to the hospital. There were 6000 patients referred to the TTP. Of the 6000 adult smokers, 985 completed our REDCap follow-up survey and acknowledged being current smokers at the time of admission to the hospital. During the study period, patients referred to the TTP were randomly assigned to one of two treatment options: 1) Enhanced care (EC), which consisted of a Tobacco Treatment Specialist (TTS) bedside consultation plus an automated post-discharge follow-up call designed to connect those smoking to the SCTQ; or 2) Basic care (BC), which consisted of the post-discharge follow-up call only. The bedside consultation consisted of an interview with a TTS to assess the patient’s smoking history, prior quitting attempts, and nicotine withdrawal symptoms. The TTS provided counseling to stop smoking, arranged for nicotine replacement therapy (NRT), and provided resources to aid in smoking cessation. Assignment to treatment conditions did not guarantee patients would actually receive the treatment offered (i.e. unavailable for bedside consultation). This study received ethics clearance through the Medical University of South Carolina IRB Committee (IRB# Protocol #107000) with a HIPAA Waiver of Authorization for Research, in which approval was granted for limited protected health information (PHI) in the EHR.

To conduct this secondary data analysis, we linked data from patients’ EHR, a REDCap telephone follow-up survey conducted six weeks after patients had been discharged back to their home, and TTP records data. The EHR data included date and time of admission, smoking status at time of admission, clinical covariates (i.e. length of stay, Charlson comorbidity index summary score, and comorbidities such as alcohol and other substance use disorders, chronic obstructive pulmonary disease [COPD], hypertension, neurological disorders, peripheral vascular diseases, among others), and demographics of our patients (i.e. age, gender, race, insurance status). The REDCap telephone follow-up survey six weeks post-discharge asked about the patient’s past smoking history (i.e. duration of smoking and age of first smoking purchase), smoking status since being discharged from the hospital, and the use of stop smoking medications since being discharged from the hospital. Lastly, TTP data included the intervention that the patient received (i.e. received bedside consultation, follow-up phone call after discharge).

### Dependent variable - smoking abstinence

Self-reported smoking abstinence at the time of the follow-up call at 6 weeks was the primary dependent variable. Patients were asked if they had smoked a cigarette, even a puff, since being discharged. Patients who answered ‘Yes’ were considered to have relapsed back to smoking, and patients who answered ‘No’ were considered smoking abstinent since their discharge from the hospital.

### Predictors of smoking abstinence

Predictors of smoking abstinence in this study fall into four categories: 1) patient demographics; 2) clinical factors related to a patient’s medical condition and hospital stay; 3) exposure to different elements of the TTP; and 4) self-reported use of stop smoking medications after discharge from the hospital. Each category of predictor variables is described below.

*Patient demographics*

The patient demographics we used were patients’ age, sex assigned at birth (male, female), race (White, Non-White), insurance status (insured, uninsured), and veteran status (veteran, not a veteran). Racial identities were dichotomized, given small cell sizes for some racial backgrounds (White, Non-White). We also used patients’ zip codes to assign them a social vulnerability score based on socioeconomic factors such as poverty, crowded housing, and lack of access to transportation. This score ranges from 0 to 1, with 0 being the least socially vulnerable and 1 being the most socially vulnerable^21^.

We also considered information about the patient’s smoking history obtained in the REDCap survey, which included information on duration of smoking and age of smoking initiation. The question on the duration of smoking asked patients to report how many years altogether they had smoked cigarettes. Patients were also asked to report at what age they first purchased cigarettes for themselves (<15, 15–17, 18–21, >21 years).

*Clinical factors related to a patient’s medical condition and hospital stay*

Clinical factors considered included the patient’s length of stay in the hospital, Charlson score (ranging from 0 to 13, with higher scores indicating higher risk of mortality)^22^, and various comorbidities determined from patients’ ICD-10 codes^23^. These comorbidities included alcohol use disorder, cardiac arrhythmia, congestive heart failure, chronic obstructive pulmonary diseases (COPD), coagulopathy, deficiency anemia, depression, diabetes, drug use disorder, fluid/ electrolyte disorder, hypothyroidism, liver disease, obesity, other neurological disorders, pulmonary circulation disorder, peripheral vascular disorder, renal failure, tumor, vascular disorder, weight loss, and pneumonia.

*Exposure to different elements of the TTP*

To be consistent with our prior study evaluating the moderating impact of exposure to the TTP, we classified patients as exposed to different levels of the service (i.e. high, medium, and any exposure) versus no exposure^13,14^. Patients who received both the bedside consultation and automated post-discharge follow-up call were considered to have had high exposure to the TTP. Patients who received exposure to either the bedside consultation or the post-discharge follow-up call, but not both, were considered to have had medium exposure. Patients who received either high or medium exposure were also considered to have had any exposure to TTP. Patients who received neither the consultation nor the follow-up call were considered to have had no exposure.

*Use of smoking cessation medications after discharge from the hospital*

In the REDCap telephone survey, we asked patients to report on their use of any smoking cessation medications (i.e. NRT such as gum, lozenge, inhaler, nasal spray, patch; varenicline/Chantix, and bupropion/Wellbutrin or Zyban) since they had been discharged from the hospital. A dichotomous smoking cessation medication use variable was created (used medication, did not use medication).

### Statistical analysis

All analyses were conducted using SAS 9.4 (Cary, NC). Descriptive statistics included frequencies and percentages for categorical variables, and means and ranges for continuous variables. Logistic regression models were used to produce unadjusted bivariate odds ratios (ORs) and adjusted odds ratios (AORs). In the adjusted multivariate regression analyses of individual predictor variables, all other variables were considered as covariates in the adjusted models. A log transformation was used to address skewness for three variables: length of stay (days), the Charlson comorbidity score, and the social vulnerability index^24^. Missing data on the predictor variables were relatively rare; therefore, we chose to exclude patients with missing data from our analyses rather than do imputation.

In an effort to isolate the impact of exposure to the different elements of the TTP on smoking abstinence, we used propensity score balancing to adjust for patient characteristics and clinical covariates^25,26^. We used the inverse probability treatment weighted (IPTW) propensity score method for balancing exposure groups on available covariates. The propensity score balancing analysis produced weights used to balance the covariates between exposure groups. The distribution of covariates between exposure group conditions was assessed unadjusted and then reassessed using the inverse probability (propensity) weights generated by the propensity model. The success of the balancing was assessed by comparing the unadjusted group differences on each covariate against those differences observed after applying propensity score weights that were used to balance the groups. Differences were tested using chi-squared analyses for categorical variables and two-sample t-tests for continuous variables.

Covariates that were not successfully balanced with a p<0.25 were then evaluated in terms of their relationship with exposure to the TTP. Covariates that still had a p<0.25 in the individual assessments were included in a weighted logistic regression model to assess the association between smoking status and exposure to the TTP. Covariates that did not reach the p<0.25 cutoff were removed, and the model was re-fit in a stepwise fashion to create a parsimonious model. Supplementary file Tables 1a–1d show the effectiveness of the propensity score balancing of covariates for our four different TTP exposure condition comparisons. Only one model, comparing enhanced versus basic care conditions (Model D), required covariates to be included in the logistic model after propensity balancing weights were applied. The added covariates in the weighted logistic model are bolded in Supplementary file Table 1d.

## RESULTS

Table 1 provides descriptive information about the study population along with unadjusted and adjusted odds ratios between self-reported smoking abstinence and predictor variables. The average age of the study sample was 53.7 years (range: 18–94); 44.7% were female; 57.9% were White, and 10.0% had no medical insurance. The average length of stay in the hospital was 4.9 days, and when log transformed, it was 1.08 days (range: 0–3.93). The average Charleson score was 1.6, and when log transformed, the mean was 0.72 (range: 0–2.48). Patients had a wide range of clinical diagnoses, 23.2% had a diagnosis of diabetes, 54.6% had hypertension, 15.9% were diagnosed with congestive heart failure, 27.8% had a diagnosis of COPD, 13.1% had a diagnosis of depression, 11.5% had a diagnosis of alcohol abuse disorder, and 12.2% had a diagnosis of other drug abuse. Overall, 70.9% of the study population were exposed to at least one element of the TTP during their hospital stay, 23.0% reported using stop smoking medications after discharge from the hospital, and 21.1% reported continuous smoking abstinence 6 weeks after discharge from the hospital.

**Table 1:** Unadjusted and adjusted odds ratios between self-reported smoking abstinence among adult current smokers followed over 6 weeks after discharge from MUSC ^a^ affiliated hospitals, March–December 2021 (N=985)

*Variables*	Total* *n (%)*	*Smoking abstinence from time of discharge to follow-up* *at 6 weeks#*		
		*n/N (%)*	*OR (95% CI)*	*AOR (95 % CI)*
**Total**		208/985 (21.1)		
**Medication use post-discharge**				
Yes (ref.)	227 (23.0)	34/227 (23.0)	1	1
No	758 (77.0)	170/758 (20.5)	0.86 (0.57–1.31)	0.86 (0.55–1.35)
**Patient characteristics**				
**Age** (years), mean (range)	985 (100)	54.2 (18–86) overall mean 53.7 (18–94)	1.00 (0.99–1.01)	**1.02 (1.00–1.04)**
**Gender**				
Female (ref.)	450 (45.7)	83/450 (18.4)	1	1
Male	535 (54.3)	125/535 (23.4)	1.35 (0.99–1.84)	**1.47 (1.01–2.14)**
**Race**				
White (ref.)	570 (57.9)	104/570 (18.2)	1	1
Non-White	415 (42.1)	104/415 (25.1)	**1.50 (1.10–2.04)**	1.40 (0.97–2.01)
**Insurance**				
Yes (ref.)	887 (90.0)	184/887 (20.7)	1	1
No	98 (10.0)	24/98 (24.5)	1.24 (0.76–2.02)	1.12 (0.63–1.98)
**Veteran status** (ever)				
Yes (ref.)	80 (8.1)	18/80 (22.5)	1	1
No	905 (91.9)	190/905 (21.0)	0.91 (0.53–1.58)	1.04 (0.56–1.95)
**Log-transformed social vulnerability index** (SVI), mean (range)	975 (99.0)	0.31 (0.0–0.69) overall mean 0.30 (0.0–0.69)	1.43 (0.68–3.00)	1.67 (0.73–3.82)
**Years smoked, mean** (range)	971 (98.6)	31.4 (2–65) overall mean 33.2 (1–70)	0.99 (0.98–1.00)	**0.97 (0.95–0.99)**
**Age 1st purchased cigarettes** (years)	979 (99.4)			
<15 (ref.)	152 (15.4)	26/152 (17.1)	1	1
15–17	279 (28.3)	54/279 (19.3)	1.16 (0.69–1.95)	0.98 (0.56–1.70)
18–21	377 (38.3)	78/377 (20.7)	1.26 (0.77–2.06)	0.89 (0.50–1.56)
>21	171 (17.4)	48/171 (28.1)	**1.89 (1.10–3.24)**	1.04 (0.52–2.10)
**Clinical characteristics**				
**Log-transformed length of hospital stay** (days), mean (range)	984 (99.9)	1.26 (0–2.94) overall mean 1.08 (0–3.93)	**1.50 (1.22–1.84)**	**1.42 (1.10–1.82)**
**Log-transformed Charlson comorbidity score,** mean (range)	985 (100)	0.73 (0–2.48) overall mean 0.72 (0–2.48)	1.04 (0.82–1.32)	1.24 (0.76–2.02)
**Alcohol use disorder**	950 (96.4)			
No (ref.)	837 (85.0)	179/837 (21.4)	1	1
Yes	113 (11.4)	20/113 (17.7)	0.79 (0.47–1.32)	0.75 (0.41–1.35)
**Cardiac arrhythmia**	950 (96.4)			
No (ref.)	805 (81.7)	164/805 (20.4)	1	1
Yes	145 (14.7)	35/145 (24.1)	1.24 (0.82–1.89)	1.04 (0.64–1.70)
**Congestive heart failure**	950 (96.4)			
No (ref.)	803 (81.5)	165/803 (20.5)	1	1
Yes	147 (14.9)	34/147 (23.1)	1.16 (0.77–1.77)	0.91 (0.53–1.57)
**Chronic obstructive pulmonary disease Dx**	950 (96.4)			
No (ref.)	676 (68.6)	146/676 (21.6)	1	1
Yes	274 (27.8)	53/274 (19.3)	0.87 (0.61–1.24)	0.85 (0.54–1.36)
**Coagulopathy Dx**	950 (96.4)			
No (ref.)	909 (92.2)	186/909 (20.5)	1	1
Yes	41 (4.2)	13/41 (31.7)	1.80 (0.92–3.55)	1.87 (0.56–4.06)
**Deficiency anemia Dx**	950 (96.4)			
No (ref.)	920 (93.4)	191/920 (20.8)	1	1
Yes	30 (3.0)	8/30 (26.7)	1.39 (0.91–3.17)	1.35 (0.53–3.44)
**Depression Dx**	950 (96.4)			
No (ref.)	821 (83.3)	175/821 (21.3)	1	1
Yes	129 (13.1)	24/129 (18.6)	0.84 (0.52–1.35)	0.95 (0.55–1.62)
**Diabetes Dx**	950 (96.4)			
No (ref.)	721 (73.2)	155/721 (21.5)	1	1
Yes	229 (23.2)	44/229 (19.2)	0.87 (0.60–1.26)	0.63 (0.38–1.04)
**Drug use disorder**	950 (96.4)			
No (ref.)	830 (84.3)	185/830 (22.3)	1	1
Yes	120 (12.1)	14/120 (11.7)	**0.46 (0.26–0.82)**	**0.37 (0.20–0.70)**
**Fluid/electrolyte disorder**	950 (96.4)			
No (ref.)	680 (69.0)	136/680 (20.0)	1	1
Yes	270 (27.4)	63/270 (23.3)	1.22 (0.87–1.71)	0.99 (0.67–1.48)
**Hypertension Dx**	950 (96.4)			
No	422 (42.8)	88/422 (20.4)	1	1
Yes	528 (53.6)	113/528 (21.4)	1.06 (0.78–1.46)	0.98 (0.66–1.46)
**Hypothyroidism**	950 (96.4)			
No (ref.)	896 (90.9)	186/896 (20.8)	1	1
Yes	54 (5.5)	13/54 (24.1)	1.21 (0.63–2.31)	1.03 (0.47–2.23)
**Liver disease Dx**	950 (96.4)			
No (ref.)	884 (89.7)	188/884 (21.3)	1	1
Yes	66 (6.7)	11/66 (16.7)	0.74 (0.38–1.44)	0.56 (0.24–1.29)
**Obesity Dx**	950 (96.4)			
No (ref.)	860 (87.3)	182/860 (21.2)	1	1
Yes	90 (9.1)	17/90 (18.9)	0.87 (0.50–1.51)	0.86 (0.46–1.59)
**Other neurological disorder**	950 (96.4)			
No (ref.)	865 (87.8)	172/865 (19.9)	1	1
Yes	85 (8.6)	27/85 (31.8)	**1.88 (1.15–3.05)**	1.59 (0.94–2.69)
**Pulmonary circulation disorder**	950 (96.4)			
No (ref.)	917 (93.1)	191/917 (20.8)	1	1
Yes	33 (3.3)	8/33 (24.2)	1.22 (0.54–2.74)	1.04 (0.40–2.68)
**Peripheral vascular disorder**	950 (96.4)			
No (ref.)	866 (87.9)	183/866 (21.1)	1	1
Yes	84 (8.5)	16/84 (19.1)	0.88 (0.50–1.55)	0.74 (0.38–1.43)
**Renal failure Dx**	950 (96.4)			
No (ref.)	863 (87.6)	175/863 (20.3)	1	1
Yes	87 (8.8)	24/87 (27.6)	1.50 (0.91–2.47)	1.10 (0.57–2.13)
**Tumor Dx**	950 (96.4)			
No (ref.)	912 (92.6)	193/912 (21.2)	1	1
Yes	38 (3.8)	6/38 (15.8)	0.70 (0.29–1.69)	0.52 (0.18–1.46)
**Vascular Disorder**	950 (96.4)			
No (ref.)	905 (91.8)	186/905 (20.5)	1	1
Yes	45 (4.6)	13/45 (28.9)	1.57 (0.81–3.05)	1.41 (0.67–2.97)
**Weight loss**	950 (96.4)			
No (ref.)	889 (90.2)	184/889 (20.7)	1	1
Yes	61 (6.2)	15/61 (24.6)	1.25 (0.68–2.29)	1.05 (0.53–2.07)
**Pneumonia Dx**				
No (ref.)	954 (96.9)	198/954 (20.7)	1	1
Yes	31 (3.1)	10/31 (31.3)	1.82 (0.84–3.92)	1.65 (0.70–3.89)

AOR: adjusted odds ratio. AORs for all other predictor variables included in the table.

a MUSC affiliated hospital contributing patients to the study were located in: Charleston, Florence, Marion, Lancaster and Chester SC (see reference #18).

* Persons with missing data are excluded from bivariate and multivariate analyses.

As shown in Table 1, while there are some differences, mostly the unadjusted bivariate odds ratios (ORs) mirror the results of the adjusted odds ratios (AORs). In the adjusted model, significant predictors of smoking abstinence included being male (AOR=1.5; 95% CI: 1.0–2.1), older age (AOR=1.02; 95% CI: 1.0–1.04), reporting fewer years of smoking (AOR=0.97; 95% CI: 0.95–0.99), having a longer length of stay in the hospital (AOR=1.42; 95% CI: 1.1–1.8), and having a drug use disorder (AOR=0.37; 95% CI: 0.2–0.7).

Table 2 shows the adjusted odds ratios between self-reported smoking abstinence and exposure to different elements of the TTP after adjusting for covariates. After adjustment for covariates, none of the odds ratios was statistically significant at the p<0.05 level.

**Table 2:** The impact of exposure to components of the tobacco treatment program (TTP) on smoking abstinence after hospital discharge, March–December 2021 (N=985)

*Intervention*	*Unadjusted smoking abstinence rate* *n (%)*	*AOR*	*95% CI*
**Model A**		0.92	0.58–1.44
High exposure^a^ to the TTP (n=209)	46 (22.0)		
vs	vs		
No exposure to the TTP (n=287)	64 (22.3)		
**Model B**		0.92	0.64–1.31
Medium exposure^b^ to the TTP (n=489)	98 (20.0) vs		
vs			
No exposure to the TTP (n=287)	64 (22.3)		
**Model C**		0.91	0.66–1.25
Any exposure^c^ to the TTP (n=698)	144 (20.6)		
vs	vs		
No exposure to the TTP (n=287)	64 (22.3)		
**Model D**		1.29	0.86–1.93
Enhanced care^d^ (n=732)	162 (22.1)		
vs	vs		
Basic care (n=253)	46 (18.2)		

AOR: adjusted odds ratio. AORs for each exposure condition are adjusted for the covariates shown in Supplementary file Tables 1a–1d. In Models A–C, after propensity score balancing, there were no significant covariates to be adjusted for. In Model D after propensity score balancing, the following covariates were used in the weighted logistic model: race, veteran status, length of stay (log transformed), alcohol abuse disorder, coagulopathy, deficiency anemia, and pneumonia. After stepwise deletion of all non-significant covariates, only race and length of stay (log transformed) remained in the model.

a High exposure to TTP is defined as receiving both the bedside consultation and post-discharge follow-up call with an option for referral to the South Carolina Quitline.

b Medium exposure to TTP is defined as receiving either the bedside consultation only or post-discharge follow-up call only, but not both.

c Any exposure to TTP is defined as A (High exposure) or B (Medium exposure).

d Enhanced care is defined as assigned to be eligible for both the bedside consultation and post-discharge call and referral to the South Carolina Quitline, whereas Basic care includes those eligible only for the post-discharge call and referral to the South Carolina Quitline.

## DISCUSSION

Patients who had more serious medical conditions necessitating a longer length of stay in the hospital were more likely to report continuous smoking abstinence after discharge from the hospital, which is consistent with other studies^18,20^. Also, consistent with previous research, being male was associated with higher levels of smoking abstinence, and a diagnosed drug use disorder was associated with lower rates of smoking abstinence^20,27-29^.

Exposure to the inpatient TTP was not associated with higher rates of continuous smoking abstinence after hospital discharge, even after adjustments for a wide range of patient characteristics and clinical covariates. Other studies have reported higher quit rates following inpatient smoking cessation counseling when combined with access to smoking cessation medications after discharge^3-14^. However, in our study, only about 23% reported using any smoking cessation medication in the post-discharge period, and only 15% overall reported getting medications prescribed to them at the time of hospital discharge. The low utilization of stop smoking medication in the post-discharge period may account for the relatively high rate of smoking (~79%) and explain why exposure to the TTP in this study was unrelated to smoking abstinence^18^. A recent study found that an EHR hard-stop prompt to clinicians prescribing smoking cessation medications to patients at the time of admission and discharge significantly increased medication use by patients and post-discharge quit rates^30^. Adopting such an intervention may be prudent to bolster the use of stop smoking medications, which can improve smoking abstinence rates after hospital discharge.

Among patients who reported that they were still smoking in our follow-up survey at 6 weeks, 43% expressed interest in receiving additional cessation assistance^18^. Thus, it may be that exposure to TTP services while inpatient served as an entry point for treatment and increased openness to engaging with such services, reinforcing the benefits of using the hospital admission and the immediate post-discharge period as opportunities to support patients in their journey towards smoking cessation^18^. Our previous studies have also shown that exposure to the TTP decreases hospital readmissions, an especially important finding in today’s environment of diminishing healthcare resources^13,14^. Indeed, hospital administrators may find this one of the most important aspects of implementing a TTP in their healthcare system. Finding efficient ways to connect patients who return to smoking after hospitalization to treatment resources is an area that is ripe for future investigation.

### Limitations

There are several limitations worth noting when interpreting current study results, including that smoking status was self-reported and not biochemically verified, which may have resulted in an overestimation of abstinence rates^31^. Indeed, self-reported smoking abstinence could introduce information bias (e.g. social-desirability bias) and misclassification bias. Additionally, residual confounding due to failure to adjust for unmeasured confounders could lead to over- or under-estimation of true associations. For example, the study did not collect information about important cessation predictors such as nicotine dependence, number of cigarettes smoked per day, past efforts to quit smoking, and the use of other nicotine-containing tobacco products (i.e. e-cigarettes, nicotine pouches). It may be the case that patients did make reductions in their tobacco use post-discharge, but this information was not captured. Also, while having standardized data on a patient’s post-discharge smoking status is a strength of this study because such data are rarely collected, the sample size available for measuring smoking abstinence was relatively modest compared to the large number of predictor variables examined, which can lead to unreliable interpretations of associations. Some cell sizes for diagnoses were small and may preclude us from reliably testing associations. Thus, we would urge readers to be cautious in interpreting the findings of reported associations.

## CONCLUSIONS

Approximately 79% of patients reported that they were smoking cigarettes again 6 weeks after discharge from the hospital. Patients requiring longer hospitalizations were more likely to report smoking abstinence after discharge.

## Supplementary Material



## Data Availability

The data supporting this research are available from the authors on reasonable request.
