# Supplementary material for: Prospective study of predictors of continuous smoking abstinence after hospital discharge
Source: Tob Prev Cessat. 2026 Apr 20;12:10.18332/tpc/217012. doi: 10.18332/tpc/217012 (PMC13114024; doi:10.18332/tpc/217012)
Supplement: Supplementary file 1 [file TPC-12-21-s1.pdf]

Supplemental Table 1a-d. The success of propensity scoring balancing of patient characteristics, co-morbidities, and medical procedures for different tobacco treatment program intervention levels (models A-C) and randomization groups (model D).

A. High intervention versus no intervention

	Unweighted			Weighted		
	High intervention (n=209)	No intervention (n=287)	p-value	High intervention	No intervention	p-value
Age (mean)	54.4	51.6	0.03	52.1	52.6	0.71
Sex<br>• Female<br>• Male	 90 (43.1%)<br>119 (56.9%)	 136 (47.4%)<br>151 (52.6%)	 0.34	 45.8%<br>54.2%	 45.1%<br>54.9%	 0.87
Race<br>• White<br>• Non-White	 119 (56.9%)<br>90 (43.1%)	 160 (55.7%)<br>127 (44.3%)	 0.79	 57.8%<br>42.2%	 57.3%<br>42.7%	 0.92
Insurance<br>• Insured<br>• Uninsured	 189 (90.4%)<br>20 (9.6%)	 257 (89.5%)<br>30 (10.5%)	 0.75	 91.1%<br>8.9%	 90.3%<br>9.7%	 0.76
Veteran<br>• Yes<br>• No	 19 (9.1%)<br>190 (90.9%)	 18 (6.3%)<br>269 (93.7%)	 0.24	 6.7%<br>93.3%	 7.4%<br>92.6%	 0.80
Log transformed social vulnerability index (SVI)	0.32	0.28	0.01	0.30	0.30	0.99
Years smoked (mean)	34.5	30.7	0.01	31.7	32.0	0.84
First purchase years<br>• <15 years<br>• 15-17 years<br>• 18-21 years<br>• ≥21 years	 39 (18.7%)<br>58 (27.9%)<br>80 (38.5%)<br>31 (14.9%)	 36 (12.7%)<br>78 (27.5%)<br>124 (43.7%)<br>46 (16.2%)	 0.28	 14.5%<br>27.5%<br>42.7%<br>15.3%	 14.8%<br>27.7%<br>42.1%<br>15.4%	 0.99
**Diagnosed Medical Conditions**						
Log transformed length of hospital stay	1.1	1.2	0.32	1.1	1.1	0.74
Log transformed Charlson comorbidity	0.7	0.7	0.88	0.7	0.6	0.48

score						
Alcohol Abuse<br>• Yes<br>• No	17 (8.5%)<br>183 (91.5%)	49 (17.6%)<br>230 (82.4%)	0.01	11.5%<br>88.5%	13.8%<br>86.2%	0.46
Cardiac Arrhythmia<br>• Yes<br>• No	25 (12.5%)<br>175 (87.5%)	49 (17.6%)<br>230 (82.4%)	0.13	16.0%<br>84.0%	14.8%<br>85.2%	0.71
Congestive Heart Failure<br>• Yes<br>• No	33 (16.5%)<br>167 (83.5%)	48 (17.2%)<br>231 (82.8%)	0.84	14.7%<br>85.3%	15.1%<br>84.9%	0.89
Chronic obstructive pulmonary disease<br>• Yes<br>• No	61 (30.5%)<br>139 (69.5%)	71 (25.5%)<br>208 (74.5%)	0.22	25.1%<br>74.9%	26.2%<br>73.8%	0.80
Coagulopathy<br>• Yes<br>• No	8 (4.0%)<br>192 (96.0%)	15 (5.4%)<br>264 (94.6%)	0.49	4.7%<br>95.3%	4.6%<br>95.4%	0.96
Deficiency Anemia<br>• Yes<br>• No	6 (3.0%)<br>194 (97.0%)	8 (2.9%)<br>271 (97.1%)	0.93	1.9%<br>98.1%	2.6%<br>97.4%	0.62
Depression<br>• Yes<br>• No	21 (10.5%)<br>179 (89.5%)	42 (15.0%)<br>237 (85.0%)	0.15	12.5%<br>87.5%	12.6%<br>87.4%	0.98
Diabetes<br>• Yes<br>• No	44 (22.0%)<br>156 (78.0%)	59 (21.1%)<br>220 (78.9%)	0.82	21.8%<br>78.2%	21.3%<br>78.7%	0.89
Drug Abuse<br>• Yes<br>• No	18 (9.0%)<br>182 (91.0%)	54 (19.4%)<br>225 (80.6%)	0.01	14.1%<br>85.9%	14.6%<br>85.4%	0.88
Fluid/electrolyte disorders<br>• Yes	53 (26.5%)	76 (27.2%)	0.86	24.4%	25.7%	0.76

• No	147 (73.5%)	203 (72.8%)		75.6%	74.3%	
Hypertension<br>• Yes<br>• No	 116 (58.0%)<br>84 (42.0%)	 147 (52.7%)<br>132 (47.3%)	 0.25	 53.6%<br>46.4%	 54.5%<br>45.5%	 0.84
Hypothyroidism<br>• Yes<br>• No	 11 (5.5%)<br>189 (94.5%)	 18 (6.5%)<br>261 (93.5%)	 0.67	 5.4%<br>94.6%	 5.0%<br>95.0%	 0.86
Liver disease<br>• Yes<br>• No	 9 (4.5%)<br>191 (95.5%)	 28 (10.0%)<br>251 (90.0%)	 0.03	 6.5%<br>93.5%	 7.6%<br>92.4%	 0.63
Obesity<br>• Yes<br>• No	 13 (6.5%)<br>187 (93.5%)	 32 (11.5%)<br>247 (88.5%)	 0.07	 11.4%<br>88.6%	 9.7%<br>90.3%	 0.58
Neurological Disorders<br>• Yes<br>• No	 13 (6.5%)<br>187 (93.5%)	 33 (11.8%)<br>246 (88.2%)	 0.05	 11.2%<br>88.8%	 9.9%<br>90.1%	 0.65
Pulmonary circulation disorder<br>• Yes<br>• No	 4 (2.0%)<br>196 (98.0%)	 10 (3.6%)<br>269 (96.4%)	 0.31	 2.7%<br>97.3%	 2.6%<br>97.4%	 0.94
Peripheral vascular disorders<br>• Yes<br>• No	 18 (9.0%)<br>182 (91.0%)	 25 (9.0%)<br>254 (91.0%)	 0.99	 6.5%<br>93.5%	 8.1%<br>91.9%	 0.53
Renal failure<br>• Yes<br>• No	 17 (8.5%)<br>183 (91.5%)	 26 (9.3%)<br>253 (90.7%)	 0.7571	 7.5%<br>92.5%	 8.1%<br>91.9%	 0.81
Tumor<br>• Yes<br>• No	 9 (4.5%)<br>191 (95.5%)	 6 (2.2%)<br>273 (97.8%)	 0.15	 2.7%<br>97.3%	 3.0%<br>97.0%	 0.85
Vascular disorders<br>• Yes<br>• No	 8 (4.0%)<br>192 (96.0%)	 14 (5.0%)<br>265 (95.0%)	 0.60	 5.3%<br>94.7%	 4.4%<br>95.6%	 0.64

Weight Loss<br>• Yes<br>• No	 18 (9.0%)<br>182 (91.0%)	 19 (6.8%)<br>260 (93.2%)	 0.38	 6.4%<br>93.6%	 7.2%<br>92.8%	 0.73
Pneumonia<br>• Yes<br>• No	 9 (4.3%)<br>200 (95.7%)	 9 (3.1%)<br>278 (96.9%)	 0.49	 2.8%<br>97.2%	 3.6%<br>96.4%	 0.64

B. Medium intervention versus no intervention

	Unweighted			Weighted		
	Medium intervention (n=489)	No intervention (n=287)	p-value	Medium intervention	No intervention	p-value
Age (mean)	54.7	51.6	0.01	53.5	53.6	0.94
Sex<br>• Female<br>• Male	 224 (45.8%)<br>265 (54.2%)	 136 (47.4%)<br>151 (52.6%)	0.67	 46.1%<br>53.9%	 46.8%<br>53.2%	0.86
Race<br>• White<br>• Non-White	 291 (59.5%)<br>198 (40.5%)	 160 (55.7%)<br>127 (44.3%)	0.31	 59.0%<br>41.0%	 58.2%<br>41.8%	0.82
Insurance<br>• Insured<br>• Uninsured	 441 (90.2%)<br>48 (9.8%)	 257 (89.5%)<br>30 (10.5%)	0.78	 90.2%<br>9.8%	 90.3%<br>9.7%	0.95
Veteran<br>• Yes<br>• No	 43 (8.8%)<br>446 (91.2%)	 18 (6.3%)<br>269 (93.7%)	0.21	 8.0%<br>92.0%	 7.3%<br>92.7%	0.73
Log transformed social vulnerability index (SVI)	0.3	0.3	0.13	0.3	0.3	0.80
Years smoked (mean)	34.2	30.7	0.01	33.5	32.1	0.26
First purchase years<br>• <15 years<br>• 15-17 years<br>• 18-21 years<br>• ≥21 years	 77 (15.8%)<br>143 (29.4%)<br>173 (35.5%)<br>94 (19.3%)	 36 (12.7%)<br>78 (27.5%)<br>124 (43.7%)<br>46 (16.2%)	0.14	 15.0%<br>29.1%<br>38.4%<br>17.6%	 15.2%<br>28.1%<br>38.9%<br>17.8%	0.99
**Diagnosed Medical Conditions**						
Log transformed length of hospital stay	1.0	1.2	0.01	1.0	1.0	0.90
Log transformed Charleson comorbidity score	0.7	0.7	0.54	0.7	0.7	0.91

Alcohol Abuse<br>• Yes<br>• No	47 (10.0%)<br>424 (90.0%)	49 (17.6%)<br>230 (82.4%)	0.01	11.7%<br>88.3%	11.6%<br>88.4%	0.98
Cardiac Arrhythmia<br>• Yes<br>• No	71 (15.1%)<br>400 (84.9%)	49 (17.6%)<br>230 (82.4%)	0.37	15.6%<br>84.4%	16.7%<br>83.3%	0.70
Congestive Heart Failure<br>• Yes<br>• No	66 (14.0%)<br>405 (86.0%)	48 (17.2%)<br>231 (82.8%)	0.24	15.1%<br>84.9%	16.4%<br>83.6%	0.62
Chronic obstructive pulmonary disease<br>• Yes<br>• No	142 (30.2%)<br>329 (69.8%)	71 (25.5%)<br>208 (74.5%)	0.17	28.9%<br>71.1%	28.6%<br>71.4%	0.93
Coagulopathy<br>• Yes<br>• No	18 (3.8%)<br>453 (96.2%)	15 (5.4%)<br>264 (94.6%)	0.32	4.2%<br>95.8%	4.0%<br>96.0%	0.87
Deficiency Anemia<br>• Yes<br>• No	16 (3.4%)<br>455 (96.6%)	8 (2.9%)<br>271 (97.1%)	0.69	3.0%<br>97.0%	2.5%<br>97.5%	0.71
Depression<br>• Yes<br>• No	66 (14.0%)<br>405 (96.0%)	42 (15.1%)<br>237 (84.9%)	0.69	14.2%<br>85.8%	14.8%<br>85.2%	0.82
Diabetes<br>• Yes<br>• No	126 (26.7%)<br>345 (73.3%)	59 (21.1%)<br>22 (78.9%)	0.0853	24.4%<br>75.6%	25.0%<br>75.0%	0.86
Drug Abuse<br>• Yes<br>• No	48 (10.2%)<br>423 (89.8%)	54 (19.4%)<br>225 (80.6%)	0.01	12.4%<br>87.6%	13.1%<br>86.9%	0.78
Fluid/electrolyte disorders<br>• Yes<br>• No	141 (29.9%)<br>330 (70.1%)	76 (27.2%)<br>203 (72.8%)	0.43	28.5%<br>71.5%	28.7%<br>71.3%	0.96

Hypertension<br>• Yes<br>• No	265 (56.3%)<br>206 (43.7%)	147 (52.7%)<br>132 (47.3%)	0.34	54.2%<br>45.8%	54.3%<br>45.7%	1.00
Hypothyroidism<br>• Yes<br>• No	25 (5.3%)<br>446 (94.7%)	18 (6.5%)<br>261 (93.5%)	0.51	5.8%<br>94.2%	6.3%<br>93.7%	0.79
Liver disease<br>• Yes<br>• No	29 (6.2%)<br>442 (93.8%)	28 (10.0%)<br>251 (90.0%)	0.05	7.4%<br>92.6%	6.7%<br>93.3%	0.73
Obesity<br>• Yes<br>• No	45 (9.5%)<br>426 (90.5%)	32 (11.5%)<br>247 (88.5%)	0.40	10.6%<br>89.4%	11.0%<br>89.0%	0.85
Neurological Disorders<br>• Yes<br>• No	39 (8.3%)<br>432 (91.7%)	33 (11.8%)<br>246 (88.2%)	0.11	10.0%<br>90.0%	10.3%<br>89.7%	0.92
Pulmonary circulation disorder<br>• Yes<br>• No	19 (4.0%)<br>452 (96.0%)	10 (3.6%)<br>269 (96.4%)	0.76	4.0%<br>96.0%	4.4%<br>95.6%	0.77
Peripheral vascular disorders<br>• Yes<br>• No	41 (8.7%)<br>430 (91.3%)	25 (9.0%)<br>254 (91.0%)	0.90	8.7%<br>91.3%	9.0%<br>91.0%	0.89
Renal failure<br>• Yes<br>• No	44 (9.3%)<br>427 (90.7%)	26 (9.3%)<br>253 (90.7%)	0.99	9.2%<br>90.8%	9.4%<br>90.6%	0.93
Tumor<br>• Yes<br>• No	23 (4.9%)<br>448 (95.1%)	6 (2.1%)<br>273 (97.9%)	0.06	3.9%<br>96.1%	2.8%<br>97.2%	0.41
Vascular disorders<br>• Yes<br>• No	23 (4.9%)<br>448 (95.1%)	14 (5.0%)<br>265 (95.0%)	0.93	5.1%<br>94.9%	5.1%<br>94.9%	0.97

Weight Loss<br>• Yes<br>• No	 24 (5.1%)<br>447 (94.9%)	 19 (6.8%)<br>260 (93.2%)	 0.33	 5.4%<br>94.6%	 4.8%<br>95.2%	 0.72
Pneumonia<br>• Yes<br>• No	 13 (2.7%)<br>476 (97.3%)	 9 (3.1%)<br>278 (96.9%)	 0.70	 2.9%<br>97.1%	 2.8%<br>97.2%	 0.93

C. Any intervention versus no intervention

	Unweighted			Weighted		
	Any intervention (n=698)	No intervention (n=287)	p-value	Any intervention	No intervention	p-value
Age (mean)	54.6	51.6	0.01	53.8	53.8	0.93
Sex<br>• Female<br>• Male	 314 (45.0%)<br>384 (55.0%)	 136 (47.4%)<br>151 (52.6%)	 0.49	 45.4%<br>54.6%	 44.8%<br>55.2%	 0.85
Race<br>• White<br>• Non-White	 410 (58.7%)<br>288 (41.3%)	 160 (55.7%)<br>127 (44.3%)	 0.39	 58.8%<br>41.2%	 58.0%<br>42.0%	 0.81
Insurance<br>• Insured<br>• Uninsured	 630 (90.3%)<br>68 (9.7%)	 257 (89.5%)<br>30 (10.5%)	 0.73	 90.4%<br>9.6%	 90.7%<br>9.3%	 0.86
Veteran<br>• Yes<br>• No	 62 (8.9%)<br>636 (91.1%)	 18 (6.3%)<br>269 (93.7%)	 0.17	 8.2%<br>91.8%	 7.7%<br>92.3%	 0.76
Log transformed social vulnerability index (SVI)	1.0	1.2	0.01	1.1	1.0	0.67
Years smoked (mean)	34.3	30.7	0.01	33.6	32.4	0.29
First purchase years<br>• <15 years<br>• 15-17 years<br>• 18-21 years<br>• ≥21 years	 116 (16.7%)<br>201 (28.9%)<br>253 (36.4%)<br>125 (18.0%)	 36 (12.7%)<br>78 (27.5%)<br>124 (43.7%)<br>46 (16.2%)	 0.15	 15.7%<br>28.6%<br>38.6%<br>17.1%	 15.0%<br>29.5%<br>38.5%<br>17.0%	 0.99
**Diagnosed Medical Conditions**						
Log transformed length of hospital stay	1.0	1.2	0.01	1.1	1.0	0.67
Log transformed Charlson comorbidity score	0.7	0.7	0.69	0.7	0.7	0.91

Alcohol Abuse<br>• Yes<br>• No	64 (9.5%)<br>607 (90.5%)	49 (17.6%)<br>230 (82.4%)	0.01	11.6%<br>88.4%	10.7%<br>89.3%	0.69
Cardiac Arrhythmia<br>• Yes<br>• No	96 (14.3%)<br>575 (85.7%)	49 (17.6%)<br>230 (82.4%)	0.20	15.0%<br>85.0%	16.0%<br>84.0%	0.68
Congestive Heart Failure<br>• Yes<br>• No	99 (14.8%)<br>572 (85.2%)	48 (17.2%)<br>231 (82.8%)	0.34	15.5%<br>84.5%	16.2%<br>83.8%	0.78
Chronic obstructive pulmonary disease<br>• Yes<br>• No	203 (30.3%)<br>468 (36.7%)	71 (25.5%)<br>208 (74.5%)	0.14	29.2%<br>70.8%	27.7%<br>72.3%	0.61
Coagulopathy<br>• Yes<br>• No	26 (3.9%)<br>645 (96.1%)	15 (5.4%)<br>264 (94.6%)	0.30	4.2%<br>95.8%	4.0%<br>96.0%	0.85
Deficiency Anemia<br>• Yes<br>• No	22 (3.3%)<br>649 (96.7%)	8 (2.9%)<br>271 (97.1%)	0.74	3.0%<br>97.0%	2.5%<br>97.5%	0.69
Depression<br>• Yes<br>• No	87 (13.0%)<br>584 (87.0%)	42 (15.0%)<br>237 (85.0%)	0.39	13.2%<br>86.8%	13.1%<br>86.9%	0.98
Diabetes<br>• Yes<br>• No	170 (25.3%)<br>501 (74.7%)	59 (21.2%)<br>220 (78.8%)	0.17	24.3%<br>75.7%	25.3%<br>74.7%	0.72
Drug Abuse<br>• Yes<br>• No	66 (9.8%)<br>605 (90.2%)	54 (19.4%)<br>225 (80.6%)	0.01	11.7%<br>88.3%	12.1%<br>87.9%	0.88
Fluid/electrolyte disorders<br>• Yes<br>• No	194 (28.9%)<br>477 (71.1%)	76 (27.2%)<br>203 (72.8%)	0.60	28.0%<br>72.0%	26.9%<br>73.1%	0.71

Hypertension<br>• Yes<br>• No	 381 (56.8%)<br>290 (43.2%)	 147 (52.7%)<br>132 (47.3%)	 0.25	 55.5%<br>44.5%	 55.8%<br>44.2%	 0.91
Hypothyroidism<br>• Yes<br>• No	 36 (5.4%)<br>635 (94.6%)	 18 (6.5%)<br>261 (93.5%)	 0.51	 5.6%<br>94.4%	 5.8%<br>94.2%	 0.89
Liver disease<br>• Yes<br>• No	 38 (5.7%)<br>633 (94.3%)	 28 (10.0%)<br>251 (90.0%)	 0.01	 6.7%<br>93.3%	 6.0%<br>94.0%	 0.66
Obesity<br>• Yes<br>• No	 58 (8.6%)<br>613 (91.4%)	 32 (11.5%)<br>247 (88.5%)	 0.18	 9.6%<br>90.4%	 10.4%<br>89.6%	 0.68
Neurological Disorders<br>• Yes<br>• No	 52 (7.7%)<br>619 (92.3%)	 33 (11.8%)<br>246 (88.2%)	 0.04	 9.0%<br>91.0%	 9.5%<br>90.5%	 0.80
Pulmonary circulation disorder<br>• Yes<br>• No	 23 (3.4%)<br>648 (96.6%)	 10 (3.6%)<br>269 (96.4%)	 0.90	 3.5%<br>96.5%	 3.7%<br>96.3%	 0.89
Peripheral vascular disorders<br>• Yes<br>• No	 59 (8.8%)<br>612 (91.2%)	 25 (9.0%)<br>254 (91.0%)	 0.93	 8.8%<br>91.2%	 8.9%<br>91.1%	 0.93
Renal failure<br>• Yes<br>• No	 61 (9.1%)<br>610 (90.9%)	 26 (9.3%)<br>253 (90.7%)	 0.91	 8.9%<br>91.1%	 9.0%<br>91.0%	 0.97
Tumor<br>• Yes<br>• No	 32 (4.8%)<br>639 (95.2%)	 6 (2.1%)<br>273 (97.9%)	 0.06	 3.9%<br>96.1%	 4.3%<br>95.7%	 0.76
Vascular disorders<br>• Yes<br>• No	 31 (4.6%)<br>640 (95.4%)	 14 (5.0%)<br>265 (95.0%)	 0.79	 4.9%<br>95.1%	 4.8%<br>95.2%	 0.97

Weight Loss<br>• Yes<br>• No	42 (6.3%)<br>629 (93.7%)	19 (6.8%)<br>260 (93.2%)	0.75	6.1%<br>93.9%	5.2%<br>94.8%	0.56
Pneumonia<br>• Yes<br>• No	22 (3.1%)<br>676 (96.9%)	9 (3.1%)<br>278 (96.9%)	0.99	3.3%<br>96.7%	3.1%<br>96.9%	0.89

D. Enhanced versus basic care conditions

	Unweighted			Weighted		
	Enhanced Care (n=732)	Basic Care (n=253)	p-value	Enhanced Care	Basic Care	p-value*
Age (mean)	53.8	53.5	0.72	54.4	53.9	0.66
Sex<br>• Female<br>• Male	 340 (46.4%)<br>392 (53.6%)	 110 (43.5%)<br>143 (56.5%)	0.41	 47.8%<br>52.2%	 46.6%<br>53.3%	0.78
Race<br>• White<br>• Non-White	 431 (58.9%)<br>301 (41.1%)	 139 (54.9%)<br>114 (45.1%)	0.27	 62.8%<br>37.2%	 57.9%<br>42.1%	**0.20**
Insurance<br>• Insured<br>• Uninsured	 658 (89.9%)<br>74 (10.1%)	 229 (90.5%)<br>24 (9.5%)	0.78	 90.3%<br>9.7%	 90.5%<br>9.5%	0.94
Veteran<br>• Yes<br>• No	 66 (9.0%)<br>666 (91.0%)	 14 (5.5%)<br>239 (94.5%)	0.08	 13.5%<br>86.5%	 7.7%<br>92.3%	**0.03**
Log transformed social vulnerability index (SVI)	0.3	0.3	1.00	0.3	0.3	0.96
Years smoked (mean)	33.5	32.6	0.44	33.9	33.5	0.75
First purchase years<br>• <15 years<br>• 15-17 years<br>• 18-21 years<br>• ≥21 years	 119 (16.3%)<br>204 (28.0%)<br>276 (37.9%)<br>130 (17.8%)	 33 (13.2%)<br>75 (30.0%)<br>101 (40.4%)<br>41 (16.4%)	0.58	 19.6%<br>25.7%<br>36.9%<br>17.8%	 16.0%<br>28.1%<br>40.5%<br>15.4%	0.46
**Diagnosed Medical Conditions**						
Log transformed length of hospital stay	1.1	1.0	0.56	1.1	1.0	**0.24**
Log transformed Charlson comorbidity score	0.7	0.7	0.98	0.7	0.7	0.96

Alcohol Abuse<br>• Yes<br>• No	92 (13.0%)<br>616 (87.0%)	21 (8.7%)<br>221 (91.3%)	0.07	18.2%<br>81.8%	10.6%<br>89.4%	**0.01**
Cardiac Arrhythmia<br>• Yes<br>• No	108 (15.2%)<br>600 (84.8%)	37 (15.3%)<br>205 (84.7%)	0.99	16.2%<br>83.8%	15.7%<br>84.3%	0.86
Congestive Heart Failure<br>• Yes<br>• No	113 (16.0%)<br>595 (84.0%)	34 (14.0%)<br>208 (86.0%)	0.48	17.8%<br>82.2%	16.1%<br>83.9%	0.57
Chronic obstructive pulmonary disease<br>• Yes<br>• No	200 (28.2%)<br>508 (71.8%)	74 (30.6%)<br>168 (69.4%)	0.49	27.9%<br>72.1%	28.2%<br>71.8%	0.93
Coagulopathy<br>• Yes<br>• No	32 (4.5%)<br>676 (95.5%)	9 (3.7%)<br>233 (96.3%)	0.60	6.1%<br>93.9%	3.8%<br>96.2%	**0.21**
Deficiency Anemia<br>• Yes<br>• No	26 (3.7%)<br>682 (96.3%)	4 (1.7%)<br>238 (98.3%)	0.12	5.9%<br>94.1%	3.2%<br>96.8%	**0.13**
Depression<br>• Yes<br>• No	96 (13.6%)<br>612 (86.4%)	33 (13.6%)<br>209 (86.4%)	0.98	12.3%<br>87.7%	15.0%<br>85.0%	0.29
Diabetes<br>• Yes<br>• No	175 (24.7%)<br>533 (75.3%)	54 (22.3%)<br>188 (77.7%)	0.45	26.5%<br>73.5%	24.8%<br>75.2%	0.63
Drug Abuse<br>• Yes<br>• No	89 (12.6%)<br>619 (87.4%)	31 (12.8%)<br>211 (87.2%)	0.92	12.0%<br>88.0%	12.1%<br>87.9%	0.98
Fluid/electrolyte disorders<br>• Yes<br>• No	200 (28.3%)<br>508 (71.7%)	70 (28.9%)<br>172 (71.1%)	0.84	28.7%<br>71.3%	26.5%<br>73.5%	0.55

Hypertension<br>• Yes<br>• No	398 (56.2%)<br>310 (43.8%)	130 (53.7%)<br>112 (46.3%)	0.5000	57.0%<br>43.0%	56.7%<br>43.3%	0.93
Hypothyroidism<br>• Yes<br>• No	37 (5.2%)<br>671 (94.8%)	17 (7.0%)<br>225 (93.0%)	0.30	4.2%<br>95.8%	4.0%<br>96.0%	0.90
Liver disease<br>• Yes<br>• No	48 (6.8%)<br>660 (93.2%)	18 (7.4%)<br>224 (92.6%)	0.73	5.9%<br>94.1%	7.1%<br>92.9%	0.51
Obesity<br>• Yes<br>• No	67 (9.5%)<br>641 (90.5%)	23 (9.5%)<br>219 (90.5%)	0.99	9.1%<br>90.9%	9.9%<br>90.1%	0.73
Neurological Disorders<br>• Yes<br>• No	68 (9.6%)<br>640 (90.4%)	17 (7.0%)<br>225 (93.0%)	0.22	11.7%<br>88.3%	9.8%<br>90.2%	0.45
Pulmonary circulation disorder<br>• Yes<br>• No	26 (3.7%)<br>682 (96.3%)	7 (2.9%)<br>235 (97.1%)	0.57	5.5%<br>94.5%	4.1%<br>95.9%	0.42
Peripheral vascular disorders<br>• Yes<br>• No	62 (8.8%)<br>646 (91.2%)	22 (9.1%)<br>220 (90.9%)	0.87	7.8%<br>92.2%	9.5%<br>90.5%	0.43
Renal failure<br>• Yes<br>• No	66 (9.3%)<br>642 (90.7%)	21 (8.7%)<br>221 (91.3%)	0.76	9.1%<br>90.9%	9.7%<br>90.3%	0.78
Tumor<br>• Yes<br>• No	27 (3.8%)<br>681 (96.2%)	11 (4.5%)<br>231 (95.5%)	0.62	3.3%<br>96.7%	3.6%<br>96.4%	0.82
Vascular disorders<br>• Yes<br>• No	35 (4.9%)<br>673 (95.1%)	10 (4.1%)<br>232 (95.9%)	0.61	5.5%<br>94.5%	5.4%<br>94.6%	0.93

Weight Loss<br>• Yes<br>• No	 46 (6.5%)<br>662 (93.5%)	 15 (6.2%)<br>227 (93.8%)	 0.87	 6.4%<br>93.6%	 6.4%<br>93.6%	 0.98
Pneumonia<br>• Yes<br>• No	 27 (3.7%)<br>705 (96.3%)	 4 (1.6%)<br>249 (98.4%)	 0.10	 6.0%<br>94.0%	 3.1%<br>96.9%	 **0.10**

*covariates with **bolded** p-values were added to propensity score weighted logistic regression model

© 2026 Roberson A. et al.
